# MVRBind: multi-view learning for RNA-small molecule binding site prediction

**DOI:** 10.1093/bib/bbaf489

**Published:** 2025-09-22

**Authors:** Song Chen, Zhijian Huang, Yucheng Wang, Yahan Li, Yaw Sing Tan, Lei Deng, Min Wu

**Affiliations:** School of Computer Science and Engineering, Central South University, Changsha 410083, China; School of Computer Science and Engineering, Central South University, Changsha 410083, China; Institute for Infocomm Research, Agency for Science, Technology and Research (A^*^STAR), Singapore 138632, Singapore; School of Computer Science and Engineering, Central South University, Changsha 410083, China; Bioinformatics Institute, Agency for Science, Technology and Research (A^*^STAR), Singapore 138671, Singapore; School of Computer Science and Engineering, Central South University, Changsha 410083, China; Institute for Infocomm Research, Agency for Science, Technology and Research (A^*^STAR), Singapore 138632, Singapore

**Keywords:** RNA-small molecule binding sites, multi-view feature fusion, multi-scale representations, apo form

## Abstract

RNA plays a critical role in cellular processes, and its dysregulation is linked to many diseases, positioning RNA-targeted drugs as an important area of research. Accurate prediction of RNA-small molecule binding sites is crucial for advancing RNA-targeted therapies. Although deep learning has shown promise in this area, challenges remain in integrating and processing multi-dimensional data, such as RNA sequences and structural features, particularly given the inherent flexibility of RNA structures. In this study, we present MVRBind, a multi-view graph convolutional network designed to predict RNA-small molecule binding sites. MVRBind generates feature representations of RNA nucleotides across different structural levels. To effectively integrate these features, we developed a multi-view feature fusion module that constructs graphs based on RNA’s primary, secondary, and tertiary structural views, enabling the model to capture diverse aspects of RNA structure. In addition, we fuse embeddings from multi-scale to obtain a comprehensive representation of RNA nucleotides, which is then used to predict RNA-small molecule binding sites. Extensive experiments demonstrate that MVRBind consistently outperforms baseline methods in various experimental settings. Our MVRBind shows exceptional performance in predicting binding sites for both the holo and apo forms of RNA, even when RNA adopts multiple conformations. These results suggest that MVRBind offers a robust model for structure-based RNA analysis, contributing toward accurate prediction and analysis of RNA-small molecule binding sites. All datasets and resource codes are available at https://github.com/cschen-y/MVRBind.

## Introduction

RNA is crucial in many cellular functions, including regulation of gene expression, catalysis, and cell signaling [1]. Due to its key involvement in various diseases, RNA has emerged as a promising drug target, especially with recent breakthroughs in RNA-targeted therapeutics. An illustrative case is the FDA-approved RNA splicing modulator risdiplam [2], which was approved in 2020 for the treatment of spinal muscular atrophy (SMA), highlighting the potential of small molecules in modulating RNA function [3]. During SMN2 pre-mRNA splicing, risdiplam promotes the highly specific inclusion of exon 7 in the mature transcript, enabling the production of sufficient functional SMN protein to compensate for the loss of SMN1 function in patients with SMA [4]. This approval has significantly increased interest in the discovery of RNA-targeted drugs. Additionally, small-molecule drugs that target RNA can substantially expand the range of druggable targets, potentially addressing diseases associated with proteins previously considered “undruggable” [5, 6]. By predicting RNA-small molecule binding sites, potential small molecules that can target the RNA of interest can be screened virtually, improving drug selectivity and speeding up the drug development process [7]. Thus, understanding RNA structure and developing reliable binding site prediction methods are essential to drive the next generation of RNA-targeted therapeutics.

While wet laboratory experiments are commonly used to identify the binding sites, they are costly and time-consuming. Computational methods have emerged as a crucial alternative, which can reduce the need for experimental screening and offer insights into RNA-small molecule interactions [8, 9]. Computational approaches for RNA binding site prediction can be classified into three categories, namely, statistical methods, traditional machine learning methods and deep learning methods. Statistical methods include Rsite [10], Rsite2 [11] and RBind [12]. Rsite identifies binding sites by calculating the extreme Euclidean distances between nucleotides in the tertiary structure of RNA [10]. Rsite2 replaces this with distances based on the secondary structure of the RNA to predict binding sites [11]. RBind constructs network properties based on the RNA’s tertiary structure and calculates the deviation between these values and their average to identify potential binding sites [12]. Among traditional machine learning methods, RNAsite derives sequence features, representing the primary structure, along with tertiary structure features, and employs random forest algorithms to predict RNA binding sites [13–15]. Rnet constructs topological properties based on the RNA’s tertiary structure and uses ensemble learning with hard voting to predict RNA binding sites [15]. Deep learning methods, such as RLBind, integrate both local and global information and employ convolutional neural networks to predict RNA binding sites [14]. Deep learning has been extensively applied in bioinformatics, as demonstrated by recent studies [16, 17]. Beyond methods specifically developed for RNA, some structure-based ligand pocket detection tools originally designed for proteins have been adapted to RNA targets. For instance, fPocket [18], a widely used protein pocket detection algorithm, has been successfully applied to RNA structures [19]. More recently, an RNA-specific version, fPocketR, has been developed to systematically identify and analyze ligand-binding pockets in RNA [20, 21]. Alongside these advances, large language models have introduced new opportunities for RNA analysis. Models including ERNIE-RNA [22], RNAErnie [23], RNABERT [24], RNA-FM [25], and UTR-LM [26] are BERT-style architectures pre-trained on extensive corpora of non-coding RNA sequences using self-supervised learning. Trained directly on raw nucleotide sequences, these models capture contextual, structural, and functional information, enabling their application to tasks like binding site prediction and druggability assessment. Their integration into RNA informatics holds promise for improving both the accuracy and generalizability of computational predictions.

Although previous methods have made progress in predicting RNA–small molecule binding sites, significant challenges remain. A major limitation is the insufficient integration of multi-view and multi-scale structural information, as well as the lack of modeling the complex relationships between them. RNA exhibits hierarchical structural organization, including primary, secondary, and tertiary levels, which collectively determine molecular binding behavior. Accurate prediction requires capturing both local and global contexts within and across these structural levels. However, most existing approaches focus on a single structural view, failing to provide a comprehensive integration of structural information. Some methods attempt to incorporate multi-scale features, but often rely on sliding windows that do not reflect the true spatial organization of RNA in two or three dimensions. These approaches also tend to overlook interactions across different views and scales. For instance, RLBind [14] models multi-scale sequence patterns but does not account for higher-order structures, while Rnet [15] incorporates structural inputs but fails to capture cross-view and cross-scale dependencies. These limitations hinder the generalization ability of models across diverse RNA conformations and reduce their robustness to noisy or incomplete structural data. Additionally, most models are trained exclusively on holo structures, while apo conformations—often the only available structural form—are largely underexplored, despite posing unique challenges for structural modeling [27].

To address these challenges, we developed MVRBind, a graph convolutional network (GCN) -based model that integrates multiple structural views and spatial scales, along with their interactions, in a unified strategy for RNA–ligand binding site prediction. First, we design a feature extraction module to generate comprehensive RNA representations across multiple views. Second, we implement a multi-view graph message passing mechanism to learn nucleotide relationships at different scales and perform cross-view feature fusion for each scale. Through multi-view learning, we can better integrate the structural information across various levels, addressing the issue of RNA’s inherent flexibility. Finally, a multi-scale prediction module is designed to effectively predict RNA-small molecule binding sites by fusing embeddings across four structural scales. Extensive experiments demonstrate that MVRBind achieves state-of-the-art performance. Meanwhile, the effectiveness and robustness of the MVRBind algorithm are validated through binding site prediction on apo RNA structures and multi-conformational RNAs.

## Materials

Five datasets (i.e. Train60, Test18, Apo test, Conformational test, and HARIBOSS) are used in our experiments. Train60 serves as the training dataset, while the three datasets (Test18, Apo test, and Conformational test) are used for testing. Specifically, Train60 and Test18 are sourced from RNAsite [13], whereas the Apo test and Conformational test datasets are curated independently. In addition, the HARIBOSS dataset [28] is split into training, validation, and test sets based on structural similarity, following the protocol of RNABind [29].

RNAsite [13] collected their dataset from the Protein Data Bank (PDB) [30] and filtered it to include only RNA chains interacting with small molecules, excluding water. A total of 1673 RNA chains were identified. Chains with lengths shorter than 20 or longer than 1500, or those containing only crystallization additives, were removed, leaving 712 RNA chains. To eliminate redundancy, they performed structural similarity clustering using the TM-score, resulting in the removal of 634 redundant structures. The final dataset consisted of 78 RNAs, which were then grouped into 57 clusters based on sequence similarity. A total of 42 out of these 57 clusters, comprising 60 RNAs with 3054 nucleotides, were designated as the training and validation set, named Train60. The Test18 set included the remaining 15 clusters with 18 RNAs and 610 nucleotides. Additionally, we randomly divided the Train60 set into training and validation sets with a 9:1 ratio.

Above, Train60 and Test18 datasets represent the holo form of RNA. To further evaluate our model’s performance in predicting binding sites for apo-form RNA and multi-conformational RNA, we constructed the Apo test and Conformational test. Specifically, we collected 10 apo-form RNA structures from the PDB and 5 apo-form RNA structures obtained through the SHAMAN method [31], resulting in 15 apo-form RNAs in total. During the redundancy removal process with the Train60 dataset, a more permissive TM-score threshold was applied to maximize the inclusion of diverse structures, ensuring a broader representation in the Apo test dataset. The specific TM-score threshold values, along with other details of the data processing steps for all test sets, are provided in the supplementary materials. This approach allowed for the retention of 8 unique RNA structures, comprising a total of 546 nucleotides, which constitute the final Apo test dataset. The PDB IDs for all RNAs are listed in the Supplementary Table S1. Furthermore, within the Apo test dataset, we identified three RNAs with 21, 20, and 6 conformations, respectively. These 47 conformations were organized into a new test set, named the Conformational test.

In addition to the four main datasets, the HARIBOSS dataset [28], which contains 353 RNA chains with 35 114 nucleotides, was also used for evaluation. In particular, following RNABind [29], the HARIBOSS dataset was preprocessed by removing RNA structures with more than 500 nucleotides and those containing modified nucleotides to ensure consistency in structural quality. The resulting dataset was clustered into 129 groups using TM-score–based structural similarity with a threshold of 0.5, following the strategy proposed in RNABind [29]. Then, four structure-based splits (i.e. Set1, Set2, Set3, and Set4, as shown in Table 1) were created as non-overlapping training, validation, and test sets based on these clusters [29]. This splitting strategy ensures that structurally similar RNAs do not appear across different sets, thereby providing a rigorous evaluation of model generalization.

**Table 1:** Details of four structure split datasets on the HARIBOSS dataset

Set	Train	Validation	Test
Set1	262 (3583:22330)	46 (565:4406)	45 (810:3420)
Set2	284 (4174:23132)	31 (350:2274)	38 (434:4750)
Set3	261 (3809:18735)	41 (376:4261)	51 (773:7160)
Set4	277 (3695:21902)	39 (675:1387)	37 (588:6867)

The first number is the RNA chain count, followed by the numbers of binding sites (positive samples) and non-binding sites (negative samples).

In this study, one binding site residue is defined as a nucleotide containing at least one atom within 4 Å of any atom in the bound ligand. For example, we have 3054 nucleotides in Train60, where 950 nucleotides are binding site residues. In the apo dataset, binding site labels are derived from the corresponding RNA holo structures that contain bound small molecules. This labeling approach follows prior work by SHAMAN [31] and RNAsite [13]. The detailed information for all the datasets above, regarding their numbers of RNA chains and nucleotides, can be found in Table 2.

**Table 2:** Dataset details

Dataset	RNA chains	Nucleotides	Pos.	Neg.
Train60	60	3054	950	2104
Test18	18	610	207	403
Apo test	8	546	76	470
Conformational test	47	1160	533	627
HARIBOSS	353	35 114	4958	30 156

## Methods

**Figure 1:**
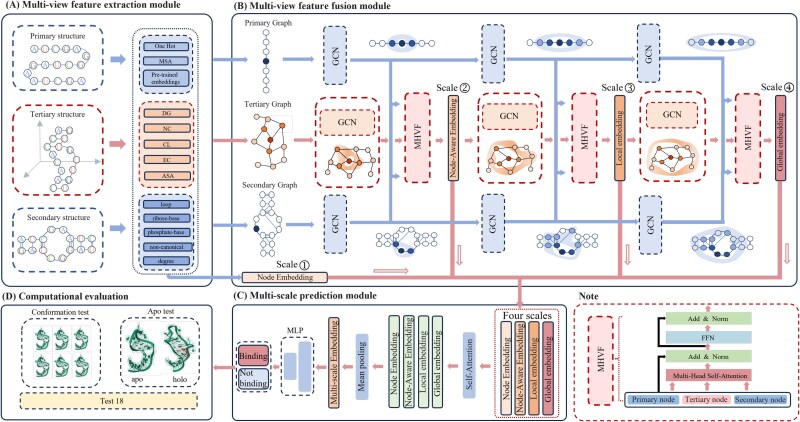
Overview of the MVRBind framework. (A) represents the multi-view feature extraction module, which is responsible for extracting nucleotide feature representations from various perspectives of the structural graph. (B) is the multi-view feature fusion module, which integrates and facilitates message passing of the features extracted from (A) through GCN and Multi-Head View Fusion (MHVF), thereby generating nucleotide representations at multiple scales. (C) is the multi-scale prediction module designed to predict RNA-small molecule binding sites based on the four-scale representations of each nucleotide derived from (A) and (B). (D) is the computational evaluation module, which combines the RNA-small molecule binding site predictions from (C) and performs the final model evaluation.

As shown in Fig. 1, our MVRBind consists of three modules, namely, the multi-view feature extraction module, the multi-view feature fusion module, and the multi-scale prediction module. In the multi-view feature extraction module, to enrich the representation of RNA nucleotides, we construct features from different structural perspectives, which provide sequential, local folding, and 3D spatial information, respectively. In the multi-view feature fusion module, we use multi-view GCN to fuse features from different views at various scales. GCNs with varying depths are employed to capture features across different scales, while graphs constructed from the primary, secondary, and tertiary structures capture nucleotide representations from different views. A multi-head attention mechanism is then applied to fuse features from different views within the same scale, allowing the model to selectively combine the most relevant information. Finally, the multi-scale prediction module integrates embeddings from four different scales using attention mechanisms, resulting in comprehensive nucleotide representations across four scales and enabling the prediction of RNA-small molecule binding sites.

### Multi-view feature extraction module

#### Primary structural features

The primary structural features are divided into three parts: the evolutionary conservation scores, the embeddings obtained by pre-training the language model RNABERT [32], and the one-hot encoding of each nucleotide residue in the RNA sequence. The multiple sequence alignment (MSA) of the researched sequence was obtained using BLASTN with an E-value threshold of 0.001, searching against a non-redundant nucleotide database. Evolutionary conservation scores for each position in the MSA were then calculated using Equation (1).


(1) \begin{align*}& \text{conservation score} = 1 - \frac{H}{H_{\text{max}}},\end{align*}

where $ H $ is the Shannon entropy [33] of a column in the MSA. Here, $ H = - \sum _{i} f_{i} \log _{2}(f_{i}) $, with $ f_{i} $ being the frequency of each nucleotide in the column, and $ H_{\text{max}} = \log _{2}(4) $ representing the maximum entropy for four possible nucleotides (A, U, G, C). This approach highlights positions with lower entropy, indicating higher conservation, and identifies evolutionarily conserved regions in the sequence. RNABERT embeddings provide compact, high-dimensional representations of RNA sequences, capturing meaningful patterns and relationships within them. Each nucleotide residue in the RNA sequence is also converted into a one-hot encoded vector as part of the preprocessing. In this encoding, each nucleotide (A, U, G, C) is represented as a binary vector of length 4, where only the corresponding position is set to 1, and all other positions are set to 0.

#### Secondary structural features

The secondary structural features of RNA were computed using the RNApdbee [34]. These features are represented as a five-dimensional binary vector, where each dimension corresponds to a specific structural characteristic: node degree (DG), base-ribose interactions, base-phosphate interactions, the presence of non-canonical base pairs, and the positioning of nucleotides within loop regions. For each of these features, except for the node DG, it is encoded as 1 if present and 0 if absent. These features are essential for accurately identifying RNA-small molecule binding sites, as they capture crucial structural information that influences binding site recognition and specificity.

#### Tertiary structural features

The tertiary structure features of RNA are divided into two main components: topological properties and solvent accessible surface areas (ASAs) of the nucleotides. The topological properties include DG, neighborhood connectivity, betweenness centrality, clustering coefficient, and eccentricity, all of which are derived from Rnet. Additionally, the ASAs of the nucleotides are computed using RNAsnap2 [14, 35].

### Multi-view feature fusion module

#### RNA nucleotides graph construction

We construct graphs for the primary, secondary, and tertiary RNA structures, where each nucleotide is represented as a node $ v_{i} $, and the feature vector $ x_{i} $ encodes the structural characteristics at the primary, secondary, and tertiary levels. The node set $ V $ represents all the nodes in the graph, typically denoted as: $ V = \{ v_{1}, v_{2}, \dots , v_{n} \} $, where $ v_{1}, v_{2}, \dots , v_{n} $ correspond to the individual nucleotides, with $ n $ being the total number of nucleotides. The feature vectors of all nucleotide nodes ($ x_{i} $, $ i =1, 2, \cdots , n$) collectively form the initial feature matrix $ X^{init} $, which is used as the input to the graph convolutional network. In the primary and tertiary structure graphs, edges connect each nucleotide to its $ k $-nearest neighbors. In the tertiary structure, distances are measured using Euclidean distance [36], while in the primary structure, distances are based on nucleotide separation in the sequence. In this study, $ k $ is set to 13 for the primary structure and 8 for the tertiary structure. The edges in the primary and tertiary structure graphs are represented by $ E_{P} $ and $ E_{T} $, respectively, indicating the connections between nucleotides in these structures. In the secondary structure graph, edges are formed between adjacent nucleotides in the backbone or those interacting through base-ribose, base-phosphate, and base-base (including non-canonical base pairs) interactions. These edges are represented by $ E_{S} $.

#### Multi-view GCN

We compute the node representations for each structural graph using a three-layer multi-view GCN and perform feature fusion across different structures using multi-head self-attention mechanisms. The outputs of the three layers of the multi-view GCN correspond to the node-aware embedding $ {Z}_{\text{NA}} $, local embedding $ {Z}_{\text{loc}} $, and global embedding $ {Z}_{\text{Glob}} $, respectively. Here, we use the computational process of the first multi-view GCN layer as an example. For each RNA, we constructed primary, secondary, and tertiary structure graphs $ G_{k} = (V, E_{k}) $, where $ k \in \{P, S, T\} $. The nucleotide representations from the GCN layer are computed as:


(2) \begin{align*}& H_{k}^{NA} = \sigma\left(\hat{A}_{k} X^{init} W_{k}^{NA}\right),\end{align*}

where $ X^{init} $ is the initial feature matrix, $ \sigma $ is the ReLU function, $ W_{k}^{NA} $ is a weight matrix and $ \hat{A}_{k} $ is the normalized adjacency matrix of $ G_{k}$. The node representations from the first GCN layer for all structural graphs $ H_{k}^{NA} $ are concatenated to form a feature matrix:


(3) \begin{align*}& {H}^{NA} = \text{Concat}\left(H_{P}^{NA}, H_{S}^{NA}, H_{T}^{NA}\right).\end{align*}

Next, we apply MHVF to $X^{NA}$ because it enables the model to simultaneously focus on different structural views of RNA, capturing complementary features across multiple subspaces. In this process, we compute the query, key, and value matrices as $Q^{NA} = X^{NA} W_{Q}$, $K^{NA} = X^{NA} W_{K}$, and $V^{NA} = X^{NA} W_{V}$, where $W_{Q}$, $W_{K}$, and $W_{V}$ are learnable weight matrices. The attention output for each head is computed as:


(4) \begin{align*}& \text{Head}_{i}^{NA} = \text{softmax}\left( \frac{Q_{i}^{NA} {K_{i}^{NA}}^{T}}{\sqrt{d}} \right) V_{i}^{NA},\end{align*}

where $ Q_{i}^{NA}, K_{i}^{NA}, V_{i}^{NA} $ are the projections for the $ i $th head, and $ d $ is the dimension of each head. The outputs of all heads are concatenated and projected linearly to obtain the multi-head attention output:


(5) \begin{align*}& \text{Attention}^{NA} = \text{Concat}\left(\text{Head}_{1}^{NA}, \dots, \text{Head}_{n}^{NA}\right) W_{O},\end{align*}

where $ n $ is the number of attention heads, and $ W_{O} $ is the projection matrix for the output. Next, we apply a feed-forward neural network (FFN) to transform the attention output:


(6) \begin{align*}& \text{FFN}^{NA} = \text{ReLU}\left(\text{Attention}^{NA} W_{1} + b_{1}\right) W_{2} + b_{2},\end{align*}

where $ W_{1}, W_{2}, b_{1}, b_{2} $ are the parameters of the feed-forward network. We then add residual connections and apply layer normalization to the output of each layer. The fusion embedding for the node-aware level is computed as:


(7) \begin{align*} {X}^{NA} &= \text{LN}\left(\text{LN}\left({H}^{NA} + \text{Attention}^{NA}\right) + \text{FFN}^{NA}\right),\end{align*}

where LN is Layer Normalization. The fused features $ {X}^{NA} $ are then used as the input to the second multi-view GCN layer for the tertiary structure, while the primary and secondary structures use graph representations obtained from the first multi-view GCN layer as input. The computation processes of the subsequent multi-view GCN layers for local and global level are similar to Equations 2 to 7. Finally, we obtain the ${X}^{NA}$, ${X}^{Loc} $, and ${X}^{Glob} $ from our multi-view GCN, containing the information from the node-aware level, local level, and global level. Then, the embeddings for the node-aware level, local level, and global level are generated through MLPs as $Z_{\text{NA}} = \text{MLP}(X^{NA})$, $Z_{\text{Loc}} = \text{MLP}(X^{Loc})$, and $Z_{\text{Glob}} = \text{MLP}(X^{Glob})$, respectively. These embeddings effectively fuse the features of the primary, secondary, and tertiary structures while preserving the unique information of each structure. Node-aware embedding captures the detailed local information about each nucleotide, focusing on its immediate structural context. Local embedding captures a broader context by considering the nucleotide’s surrounding context and local interactions, offering a more comprehensive view beyond its immediate neighborhood. Global embedding captures the high-level representation of the tertiary structure, providing a global view of the nucleotide’s context.

### Multi-scale prediction module

Through the multi-view GCN, nucleotide embeddings are obtained at multiple scales. The initial feature matrix $ X_{\text{init}} $, serving as the embedding at the smallest scale, is referred to as the initial binding feature $ Z_{\text{Node}} $ for each nucleotide. This results in a total of four scale-specific representations for each nucleotide. These four representations $ Z_{\text{Node}}, Z_{\text{NA}}, Z_{\text{Loc}}$, and $ Z_{\text{Glob}} $ are processed using self-attention to capture the relevant features at each scale. Specifically, the representations are transformed into queries $ Q $, keys $ K $, and values $ V $, and the attention scores $ A $ are computed to capture the dependencies between the different scales. The detailed operations, including query, key, and value transformations, attention score computation, and weighted summation, are provided in the supplementary material. The attention scores $ A $ are then used to compute the self-attention-enhanced representations $ Z_{\text{Node}}^{\text{a}}, Z_{\text{NA}}^{\text{a}}, Z_{\text{Loc}}^{\text{a}}, Z_{\text{Glob}}^{\text{a}} $ for the four scales, respectively. These four self-attention-enhanced representations are then combined through average pooling, integrating information from all scales into a comprehensive feature representation for each nucleotide. Specifically, the final representation is computed using average pooling over the four scales.


(8) \begin{align*}& Z_{\text{f}} = \text{AveragePooling}\left(Z_{\text{Node}}^{\text{a}}, Z_{\text{NA}}^{\text{a}}, Z_{\text{Loc}}^{\text{a}}, Z_{\text{Glob}}^{\text{a}}\right).\end{align*}

This combined multi-scale representation $ Z_{\text{f}} $ is then fed into an MLP for classification, producing the predicted output:


(9) \begin{align*}& \hat{y} = \text{MLP}(Z_{\text{f}}),\end{align*}

where $\hat{y}$ is the output of the MLP, representing the classification for all the $n$ nucleotides. The value of $ \hat{y_{i}} \in \{0, 1\}, 1\leq i \leq n $, where $ 1 $ indicates that the $ i^{\mathrm{th}}$ nucleotide is a binding site and $ 0 $ indicates that it is not. The model is trained to minimize the binary cross-entropy loss. During training, the learning rate is set to 6e-5, the batch size is 30, and the model is trained for 200 epochs. Adam is used as the optimizer.

## Results

In this section, we systematically evaluate MVRBind’s performance across different RNA states and datasets, including holo and apo datasets. All experiments were conducted on a single NVIDIA RTX 4070 GPU with 8 GB of memory. The model contains approximately 1.47 million trainable parameters, with a total size of 6.0 MB. A summary of training and inference efficiency is provided in Table 3. The total inference times are exemplified by the results on Test18 and Set1.

**Table 3:** Summary of model training and inference efficiency

Metric	Train60	HARIBOSS
Total training time	36 s	204 s
Total inference time	0.18 s (Test18)	0.59 s (Set1)
Inference time per residue	$3.0 \times 10^{-4}$ s	$1.3 \times 10^{-4}$ s

### Model evaluation and test sets

RNA–small molecule binding site prediction aims to identify which nucleotides in an RNA molecule bind to small molecules as binding sites and which do not as non-binding sites. To evaluate the performance of such prediction models, several standard classification metrics are used. In this context, true positives (TPs) are nucleotides correctly predicted as binding sites, false positives (FPs) are non-binding nucleotides incorrectly predicted as binding sites, true negatives (TNs) are non-binding nucleotides correctly predicted as non-binding, and false negatives (FNs) are binding sites incorrectly predicted as non-binding. Precision measures the proportion of predicted binding sites that are actually correct:


(10) \begin{align*}& \text{Precision} = \frac{TP}{TP + FP}.\end{align*}

Recall quantifies the proportion of actual binding sites that are successfully identified:


(11) \begin{align*}& \text{Recall} = \frac{TP}{TP + FN}.\end{align*}

The Matthews Correlation Coefficient (MCC) provides a balanced evaluation of binary classification performance, particularly under class imbalance, and is defined as:


(12) \begin{align*}& \text{MCC} = \frac{TP \cdot TN - FP \cdot FN}{\sqrt{(TP + FP)(TP + FN)(TN + FP)(TN + FN)}}.\end{align*}

The Area Under the ROC Curve (AUC) reflects the model’s ability to distinguish between binding and non-binding nucleotides across all decision thresholds; a higher AUC indicates better overall discriminative performance. Together, these metrics provide a comprehensive and robust assessment of model accuracy, reliability, and generalizability in RNA–small molecule binding site prediction.

As mentioned earlier, we evaluated the model’s performance using three datasets shown in Table 2: Test18, Apo test, and Conformational test. To assess performance across different RNA states, we categorized these datasets into holo and apo groups. The holo category includes Test18 and reflects the prediction of small molecule binding sites on RNA after ligand binding has occurred. The apo category, which includes the Apo test and Conformational test, focuses on predicting binding sites prior to ligand binding. This distinction is important because, in real-world drug development, the apo form of RNA is typically the only available structure, making strong apo performance crucial for practical applications. Moreover, the conformational test evaluates the model’s ability to handle RNA structural flexibility by testing across diverse conformations. In addition to these datasets, we conducted experiments on the HARIBOSS dataset to examine model generalization to larger and more diverse RNA structures. The dataset was processed following the clustering and splitting strategy proposed in RNABind [29].

For a comprehensive evaluation, we compared MVRBind with existing methods including Rsite [10], Rsite2 [11], RBind [12], RNAsite [13], RLBind [14], Rnet [15], and RNABind [29]. We also compared with RNA foundation models RNABERT [24], RNAErnie [23], and RNA-FM [25], which were fine-tuned by updating all their parameters on our Train60 dataset, with a multilayer perceptron classification head added for binding site prediction. These methods cover a wide range of approaches and provide a thorough benchmark to evaluate MVRBind’s performance in RNA small molecule binding site prediction.

### Performance comparison on holo dataset Test18

In order to evaluate the performance of our model for RNA small molecule binding site prediction in holo form, we compared MVRBind with other baselines on Test18. The results of various methods on Test18 are shown in Table 4.

**Table 4:** Performance comparison across different test sets

Models	Test18				Apo test				Conformational test			
	Precision	Recall	MCC	AUC	Precision	Recall	MCC	AUC	Precision	Recall	MCC	AUC
Rsite	0.394	0.126	0.040	0.513	0.224	0.171	0.085	0.538	0.465	0.131	0.027	0.509
Rsite2	0.333	0.184	0.006	0.497	0.167	0.105	0.025	0.510	–	–	–	–
RNAsite	0.550	0.159	0.147	0.710	0.368	0.092	0.126	0.703	0.508	0.263	0.007	0.592
RBind	0.611	0.159	0.179	0.554	0.224	0.145	0.077	0.532	0.569	0.145	0.068	0.519
RLBind	0.632	0.320	0.277	0.719	0.200	0.013	0.016	0.577	0.597	0.109	0.076	0.398
Rnet	0.616	0.333	0.277	0.709	0.375	0.039	0.083	0.502	0.554	0.184	0.080	0.560
RNABind	0.463	0.341	0.263	0.717	0.266	**0.228**	0.144	0.657	0.442	0.274	0.018	0.532
RNABERT+MLP	0.569	0.338	0.243	0.682	0.215	0.144	0.070	0.696	0.472	0.285	0.142	0.599
RNAErnie+MLP	0.430	0.135	0.066	0.569	0.261	0.145	0.102	0.544	0.597	0.225	0.126	0.614
RNA-FM+MLP	0.435	0.275	0.105	0.591	0.203	0.144	0.061	0.586	0.475	0.183	0.015	0.593
MVRBind (*Ours*)	**0.645**	**0.342**	**0.351**	**0.745**	**0.466**	0.184	**0.228**	**0.756**	**0.632**	**0.296**	**0.181**	**0.660**

Rsite predicts binding sites based on sequence-derived secondary structure. Since all conformations in the Conformational test set share the same RNA sequence, Rsite yields identical predictions and is therefore not applicable to this evaluation.

MVRBind outperforms other baselines across all the evaluation metrics on Test18 dataset. Specifically, it achieves a precision of 0.645, recall of 0.342, MCC of 0.351, and AUC of 0.745. Notably, MVRBind achieves improvements of 2.0% in precision, 2.7% in recall, 26.7% in MCC, and 3.6% in AUC compared to the best-performing baseline. In RNA-small molecule binding site prediction, precision ensures accurate identification of true binding sites with minimal false positives, and our model performs well in this aspect. Recall detects as many true binding sites as possible, and despite lower recall than precision, the balance shows the model captures relevant sites effectively. The 26.7% improvement in MCC indicates the model handles both positive and negative predictions well, even with imbalanced data. The AUC of 0.745 confirms that MVRBind effectively differentiates between bound and non-bound RNA nucleotides.

**Figure 2:**
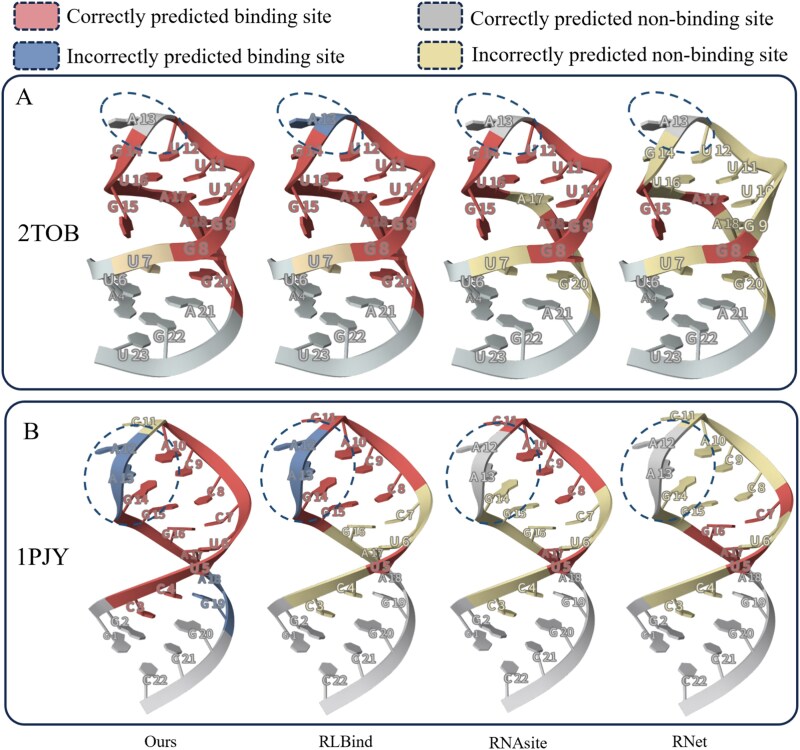
Prediction of small molecule binding sites in RNA from Test18 (PDB ID 2TOB, A) and Apo test (PDB ID 1PJY, B), showing correctly predicted binding and non-binding sites, missed binding sites, and incorrectly predicted binding sites.

Our model can accurately predict even in regions that are difficult to predict, such as those influenced by the RNA’s tertiary structure. These regions may adopt configurations that differ from the local context, complicating predictions. As illustrated in Fig. 2A, the tenth nucleotide of the RNA in Test18 (PDB 2TOB) is a non-binding site surrounded by multiple binding sites, posing a challenge for traditional methods. Sliding window-based approaches, such as RLBind, RNAsite, and Rnet, primarily rely on local contextual features. As a result, RLBind and RNAsite misclassify the tenth nucleotide as a binding site due to the strong influence of the surrounding binding residues. Although Rnet correctly identifies the tenth nucleotide as a non-binding site, this prediction is a consequence of misclassifying all surrounding binding residues as non-binding sites. This highlights the limitations of these methods in accurately distinguishing non-binding sites in complex local contexts. In these cases, if binding sites dominate the surrounding area, sliding window methods may incorrectly classify the non-binding site as a binding site. Our method, however, addresses this by incorporating multiple structural levels, enabling the model to recognize unique configurations.

### Performance comparison on apo datasets

Predicting potential small molecule binding in the apo state of RNA can provide insights for the development of new drugs [37]. Hence, we also compared MVRBind with other baselines on apo datasets as shown in Table 4, including the Apo test and Conformational test.

MVRBind excels at accurately capturing RNA-small molecule binding sites in the apo state, owing to its comprehensive feature representations obtained through multi-view GCN and self-attention mechanisms. The model effectively captures complex local interactions and global contextual features by integrating information across multiple structural views. This multi-scale feature fusion enables MVRBind to focus on the most relevant features at different scales, producing robust nucleotide embeddings. As a result, MVRBind demonstrates superior performance, particularly in terms of AUC and precision. Note that RNABind achieves slightly higher recall than MVRBind on the Apo test set with 0.228 compared to 0.184, while MVRBind demonstrates significantly better precision (0.466 versus 0.266). This leads to better overall performance, with MVRBind reaching an MCC of 0.228 and an AUC of 0.756, outperforming RNABind, which achieves 0.144 and 0.657, respectively.

Similarly, even in the apo form of RNA, MVRBind can make accurate predictions in regions prone to interference from specific contextual factors. As illustrated in the Fig. 2B, the twelfth and thirteenth nucleotides of the RNA in the Apo test (PDB 1PJY) are non-binding sites, surrounded entirely by binding sites. Despite this, MVRBind successfully predicts these non-binding sites. In contrast, while RNAsite and Rnet also predict the correct result at this position, they do so by classifying a large number of surrounding binding sites as non-binding, which leads to a shift in the overall structural pocket position. MVRBind provides a more accurate prediction of the overall position of the binding pocket. To further understand the model’s behavior, we conducted a error analysis on representative apo RNA samples. As shown in Supplementary Fig. S7, most false positives and false negatives occur in flexible internal loops, where complex tertiary folding and large cavities make precise residue-level prediction more difficult. Nonetheless, MVRBind still accurately identifies the overall binding pocket. Detailed results are provided in the Supplementary Section 7.

For the same RNA, multiple conformations may exist, which presents a challenge in predicting the binding sites of small RNA molecules. To evaluate the model’s ability to predict binding sites in different conformations, we also conducted the Conformational test on the apo form, comparing our method with other baselines. The results as shown in Table 4 demonstrate the performance of different models in the RNA-small molecule binding site prediction task. MVRBind outperforms the other models in all four metrics: precision (0.632), recall (0.296), MCC (0.181), and AUC (0.660), indicating it is the best at accurately predicting binding sites, identifying more true binding sites, and distinguishing between binding and non-binding sites. In contrast, other models such as Rsite and RLBind perform worse on these metrics.

### Binding sites prediction without experimental structures

Given the limited availability of experimentally resolved RNA tertiary structures, we utilized RNAcomposer [38] to predict structures for all the RNAs in Test18 dataset, ensuring consistency with previous methods, and performed binding site predictions. MVRBind consistently outperforms existing methods across all evaluation metrics, as demonstrated in Supplementary Table S3.

Despite structural deviations, MVRBind remains robust by preserving essential secondary structure motifs. For example, in the RNA with PDB ID: 1FMN RMSD = 7.14 Å), the binding site remains intact despite deviations at the 5$^\prime $ and 3$^\prime $ ends, enabling accurate predictions, as shown in Supplementary Fig. S2A. Certain RNAcomposer-generated structures exhibited high RMSD values (>20Å) due to the omission of interchain interactions in multi-chain RNAs. To address this, we employed AlphaFold3 [39], which provides a more comprehensive modeling approach. Even for the least accurate prediction (PDB ID: 1AJU, RMSD = 10.08 Å), secondary structure motifs were largely preserved, as shown in Supplementary Fig. S2B, ensuring reliable prediction performance. Predictions on AlphaFold3-generated structures resulted in a slight decrease in AUC (AUC = 0.73), with a drop of exactly 2% compared to predictions using experimentally resolved structures. Furthermore, as shown in Supplementary Fig. S8, our results indicate that MVRBind’s binding site prediction is insensitive to moderate deviations in predicted secondary structures, maintaining stable performance when inaccuracies remain within a reasonable range. This highlights MVRBind’s robustness in predicting binding sites for structurally flexible RNAs. Further details can be found in the Supplementary Section 8. Together, these findings support the applicability of MVRBind to computationally predicted RNA structures, as long as essential secondary structure elements are preserved and low-confidence regions are appropriately filtered using tools such as AlphaFold3.

### Performance comparison on HARIBOSS dataset

To further evaluate the robustness and generalization ability of MVRBind, we conducted experiments on the HARIBOSS dataset using four different structure-based data splits, as summarized in Table 1. We compared MVRBind with three representative baseline methods: RLBind [14], RNet [15], and RNABind [29], under identical experimental settings. These three baselines were selected for the following reasons. First, RNABind is the most recent method for RNA–ligand binding site prediction, published in 2025. Second, RLBind and Rnet demonstrated strong performance in our prior evaluation on the Test18 dataset.

**Figure 3:**
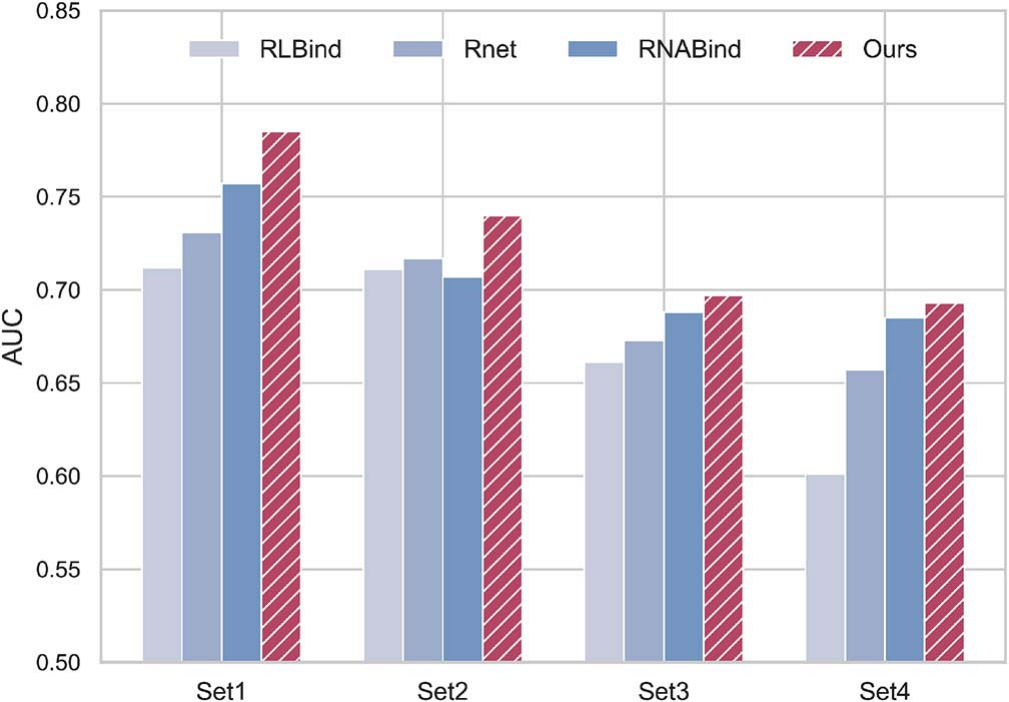
AUC scores of RNABind, RLBind, RNet, and our method evaluated on four structural splits of the HARIBOSS dataset.

As shown in Fig. 3, MVRBind consistently outperforms all baseline methods across the four structural splits. The most significant improvement is observed on the Set1, where MVRBind achieves an AUC of 0.785, outperforming the best baseline RNABind by 0.028. These results highlight the superior generalization capability of our method in structurally diverse scenarios. More detailed comparison results can be found in the Supplementary Section 9.

### Ablation study

To evaluate the contribution of each component in our model, we conducted an ablation study on Test18, Apo test, and Conformational test by testing seven different variant. The first category examines the role of structural information by removing the graph representation at each scale. Specifically, we tested MVRBind without the primary structure graph (w/o prim. str.), MVRBind without the secondary structure graph (w/o sec. str.), and MVRBind without the tertiary structure graph (w/o ter. str.). These variants assess the extent to which structural connectivity at different levels contributes to accurate binding site predictions. The second category investigates the effect of feature representations by eliminating structure-derived features. The tested variants include MVRBind without primary structure features (w/o prim. feat.), MVRBind without secondary structure features (w/o sec. feat.), and MVRBind without tertiary structure features (w/o ter. feat.). This analysis provides insights into the significance of structural embeddings at different scales. The third category evaluates the necessity of the multi-scale fusion mechanism, using a variant termed MVRBind without multi-scale fusion (w/o msf).

The results, shown in Table 5, indicate that removing any component of MVRBind reduces performance, with the largest drops occurring when the tertiary structure or multi-scale prediction module is removed, highlighting the importance of all structural features and multi-scale fusion. We also conducted the ablation study on both the Apo test and Conformational test. The results as shown in Supplementary Table S4, confirm these findings, with the full MVRBind model consistently achieving the best results.

**Table 5:** Results of ablation study on Test18

Models	Precision	Recall	MCC	AUC
MVRBind (w/o prim. str.)	0.605	0.304	0.255	0.727
MVRBind (w/o sec. str.)	0.515	0.314	0.222	0.721
MVRBind (w/o ter. str.)	0.585	0.280	0.229	0.720
MVRBind (w/o prim. feat.)	0.555	0.072	0.098	0.690
MVRBind (w/o sec. feat.)	0.639	0.188	0.211	0.690
MVRBind (w/o ter. feat.)	0.469	0.328	0.152	0.680
MVRBind (w/o msf)	0.571	0.096	0.120	0.710
**MVRBind**	**0.645**	**0.342**	**0.351**	**0.745**

### Sensitivity analysis

We conducted a sensitivity analysis to examine the impact of structural and architectural design choices on MVRBind’s performance. Specifically, we evaluated the effect of the number of $k$-nearest neighbors used in the tertiary structure graph, as well as the number of GCN layers.

**Figure 4:**
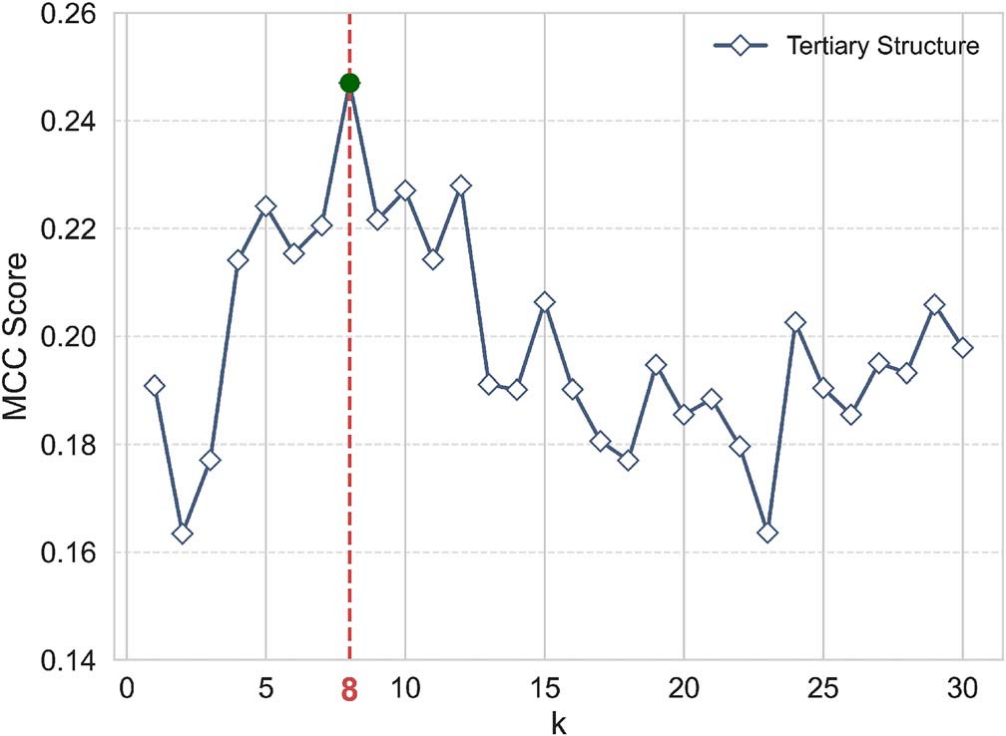
The MCC values of MVRBind were evaluated under various $k$ values in the tertiary structural graph.

As shown in Fig. 4, model performance peaks when $k$ equals 8 in the tertiary graph. Increasing the number of GCN layers initially improves performance but begins to degrade when the number exceeds 3, due to over-smoothing effects as summarized in Table 6.

**Table 6:** Performance comparison across different layers

Layers	Test18	Apo test	Conformational test
1	0.707	0.694	0.571
2	0.714	0.727	0.639
3 (Ours)	0.745	0.756	0.660
4	0.723	0.646	0.577
5	0.702	0.739	0.419
6	0.718	0.722	0.370

We also explored the impact of $k$ in the primary structure graph and compared various graph convolutional backbones. Varying $k$ from 1 to 30, we found that performance improved up to $k=13$ and then declined, Supplementary Fig. S1. We also tested alternative graph neural networks including GraphSAGE [40], GATv2 [41], k-GNNs [42], and TAGCN [43]. GCN [44] consistently performed best, likely because its simpler architecture reduces the risk of overfitting on limited data. These results are detailed in Supplementary Table S2.

### Case study

To validate the ability of MVRBind to predict RNA-small molecule binding sites in actual drug development, we predicted the binding sites for SMN2 pre-mRNA. In patients with SMA, the deficiency of the SMN1 gene leads to insufficient levels of SMN protein. Although the SMN2 gene can also produce some SMN protein, the amount is insufficient to maintain normal muscle function and morphology. Risdiplam is a drug that targets SMN2 pre-mRNA to improve the production of functional SMN protein and is the first FDA-approved oral medication for treating SMA.

**Figure 5:**
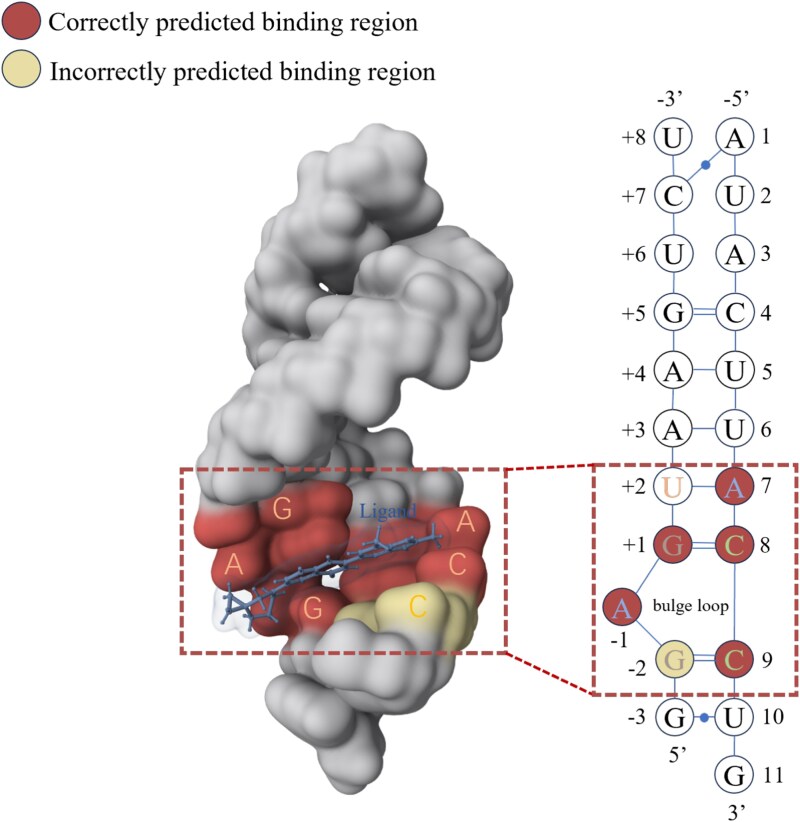
Visualization of risdiplam’s binding, illustrating the predictive results of MVRBind in conjunction with the constructed secondary structure graph, where red denotes successfully predicted parts of the ligand-binding pocket, and yellow indicates regions where the prediction did not match the known pocket.

As shown in the Fig. 5, risdiplam binds to the RNA helix formed by the 5$^\prime $ splice site of SMN2 exon 7 and the 5$^\prime $ end of U1 small nuclear RNA (snRNA), primarily stabilizing the “bulge” structure at the 5$^\prime $ splice site. Specifically, the central aromatic ring of risdiplam inserts between C8 and C9 of U1 snRNA, forming tight interactions with the RNA. Its carbonyl group forms a direct hydrogen bond with the amino group of adenine at position −1 of the RNA, while the positively charged amine in its piperazine group forms a salt bridge with the negatively charged oxygen in the phosphate group of C9. These interactions together enhance the stability of the complex. Furthermore, an HN to C9 phosphate oxygen hydrogen bond further strengthens the binding of risdiplam to the RNA [45]. We constructed the secondary graph by the tertiary structure of SMN2 pre-mRNA, which emphasize the critical role of bulged loops in secondary structures. As this region provides a structural platform that allows risdiplam to bind more effectively to RNA and stabilize the 5$^\prime $ splice site, it is the key for risdiplam binding to SMN2 pre-mRNA. By interacting with the bulge loop, risdiplam enhances the stability of this region, promoting the correct splicing of SMN2 exon 7 [46]. To eliminate potential redundancy, we removed from the training set the two RNAs with the highest structural similarity to 8R62, namely PDB IDs 1YLS and 2QUW, with TM-scores of 0.363 and 0.388 respectively, while all others were below 0.3. Sequence-level clustering using MMseqs2 [47] with a 30% identity threshold showed that no RNA in the training set shares high sequence similarity with 8R62. More results and details are provided in Supplementary Section 6. We then retrained the model and performed binding site prediction on 8R62. We visualized the predicted binding pockets in Fig. 5. The red regions indicate correctly predicted ligand-binding pockets, while the yellow region represents a mispredicted site located at the structural periphery of the known binding pocket. The results indicate that MVRBind incorporates structural information from multiple perspectives and highlights the importance of the bulge loop in the secondary structure graph. Through multi-view learning, MVBRind can better capture critical information that might be difficult to recognize from a single perspective. This information is essential for accurately predicting binding sites. This multi-view learning method allows for a deeper understanding of the binding mechanism between risdiplam and RNA, offering valuable theoretical support for drug optimization.

## Discussion and conclusion

We developed MVRBind, a GCN-based model that integrates multiple structural views and spatial scales, along with their interactions, in a unified strategy for RNA–ligand binding site prediction. The model outperforms baseline methods in both holo and apo forms, demonstrating high accuracy and robustness across RNA conformations. While MVRBind performs well, its accuracy decreases for RNAs with complex tertiary structures or interactions with other chains. Additionally, certain RNA classes such as long non-coding RNAs remain underrepresented in our dataset due to limited availability of experimentally resolved structures, which may bias the model toward well-represented RNA families and motifs. Although we removed redundant sequences and structures between training and test sets to promote generalization, the model has not been systematically evaluated on RNAs with entirely novel folds.

Future improvements may include co-folding predictions, ensemble learning, and the integration of experimental data to enhance the model’s robustness and generalizability. In particular, we aim to incorporate detailed experimental annotations, such as crystallographic and spectroscopic conditions, functional descriptions of binding sites, mutation-induced changes in binding affinity or RNA structure, and atomic-level ligand interactions. These annotations will be derived from X-ray or NMR-resolved structures and the associated literature, and are expected to help refine model inputs and improve predictive accuracy. Additionally, we plan to explore fine-tuning and domain adaptation strategies, including transfer learning and self-supervised pretraining, by leveraging MVRBind’s modular architecture to improve performance on underrepresented or novel RNA classes.

In conclusion, MVRBind provides a robust approach for structure-based analysis of RNA–ligand binding sites. Its ability to generalize across diverse RNA conformations lays a solid foundation for precise prediction and comprehensive analysis of RNA–small molecule binding sites, facilitating future progress in RNA-targeted molecular studies.

**Key Points**

- We propose MVRBind, a multi-view graph convolutional network that predicts RNA-small molecule binding sites by leveraging structural features at multiple levels.
- MVRBind constructs graphs from RNA’s primary, secondary, and tertiary structures to capture diverse structural perspectives.
- A multi-view feature fusion module integrates information across structural views and multiple scales to generate comprehensive nucleotide representations.
- Extensive experiments show that MVRBind outperforms baselines and remains effective across different RNA conformations, including both holo and apo forms.

## Supplementary Material

MVRBind_Supplementary_Material_bbaf489
