# Supplementary material for: MVRBind: multi-view learning for RNA-small molecule binding site prediction
Source: Brief Bioinform. 2025 Sep 22;26(5):bbaf489. doi: 10.1093/bib/bbaf489 (PMC12451103; doi:10.1093/bib/bbaf489)
Supplement: MVRBind_Supplementary_Material_bbaf489 [file mvrbind_supplementary_material_bbaf489.pdf]

Supplementary Materials for "MVRBind: Multi-view Learning for RNA-Small Molecule Binding Site Prediction"

Song Chen [1], Zhijian Huang [1], Yucheng Wang [2], Yahan Li [1], Yaw Sing Tan [3], Lei Deng [1,*] and Min Wu [2,*]

*To whom correspondence should be addressed.

[1]School of Computer Science and Engineering, Central South University, Changsha, 410083, China and

[2]Institute for Infocomm Research, Agency for Science, Technology and Research (A*STAR), 138632, Singapore and

[3]Bioinformatics Institute, Agency for Science, Technology and Research (A*STAR), 138671, Singapore.

Contents

1 Data processing steps **2**

2 Sensitivity analyses **3**

3 Details of self-attention application in multi-scale prediction module **4**

4 Binding sites prediction without experimental structures **5**

5 Ablation study on Apo Test and Conformational Test **8**

6 Similarity analysis between 8R62 and the training set **9**

7 Error analysis on apo RNA structures **11**

8 Robustness to predicted structure quality **12**

9 Comprehensive Evaluation Metrics on Benchmark Datasets **13**

1 Data processing steps

The Train60 and Test18 datasets were derived from the Protein Data Bank(PDB) following these steps. Initially, 1673 RNA chains associated with small molecules (excluding water) were collected. RNAs with chain lengths outside the range of 20 to 1500, those containing crystallization additives, or those lacking interactions with small molecules were excluded, leaving a total of 712 chains. Interactions were defined based on a <4 Å distance between a nucleotide and a small molecule. To reduce redundancy, pairwise structural similarity (TM-score) was calculated using RNA-align [1], resulting in 78 unique RNA chains. About 75% of these were designated for training, and the remaining 25% for testing. Sequence-based clustering was then performed, grouping the 78 RNA sequences into 57 clusters at 30% sequence identity. The training set (Train60) consists of 60 RNAs from 42 clusters, while the test set (Test18) contains 18 RNAs from 15 clusters [2, 3, 4].

Table S1: Datasets and Corresponding PDBID_chain.

**Dataset**	**PDBID_chain**
Apo test	1MME_B, 1PJY_A, 1SCL_A, 2L5Z_A, 5E54_A, 6UES_A, 7WIA_V, 8SA6_A
Conformational test	1PJY_A, 1SCL_A, 2L5Z_A

The Apo test and Conformational test datasets were constructed as follows. We collected 6 apo-form RNA structures from the Protein Data Bank and 5 apo-form RNA structures obtained through the SHAMAN method [5]. Additionally, we identified 4 apo-form RNAs from Test18, resulting in a total of 15 apo-form RNAs. To ensure a broader structural diversity in the Apo test dataset, we applied a more permissive TM-score threshold of 0.6 during the redundancy removal process with the Train60 dataset, along with a 30% sequence similarity threshold. This allowed the retention of 8 unique RNA structures, totaling 546 nucleotides, which constitute the final Apo test dataset. Furthermore, within the Apo test dataset, we identified three RNAs with distinct conformations (21, 20, and 6 conformations, respectively). These structures were organized into a new test set designated as the Conformational test. The PDB ID details of the RNA corresponding to the Apo and Conformaitonal tests are shown in the Table S1.

2 Sensitivity analyses

We varied the number of k-nearest neighbors in the primary structure graph from 1 to 30 and evaluated the Matthews Correlation Coefficient (MCC) on the validation set. As shown in Fig. S1, performance improved until $k = 13$, after which further increases degraded performance.

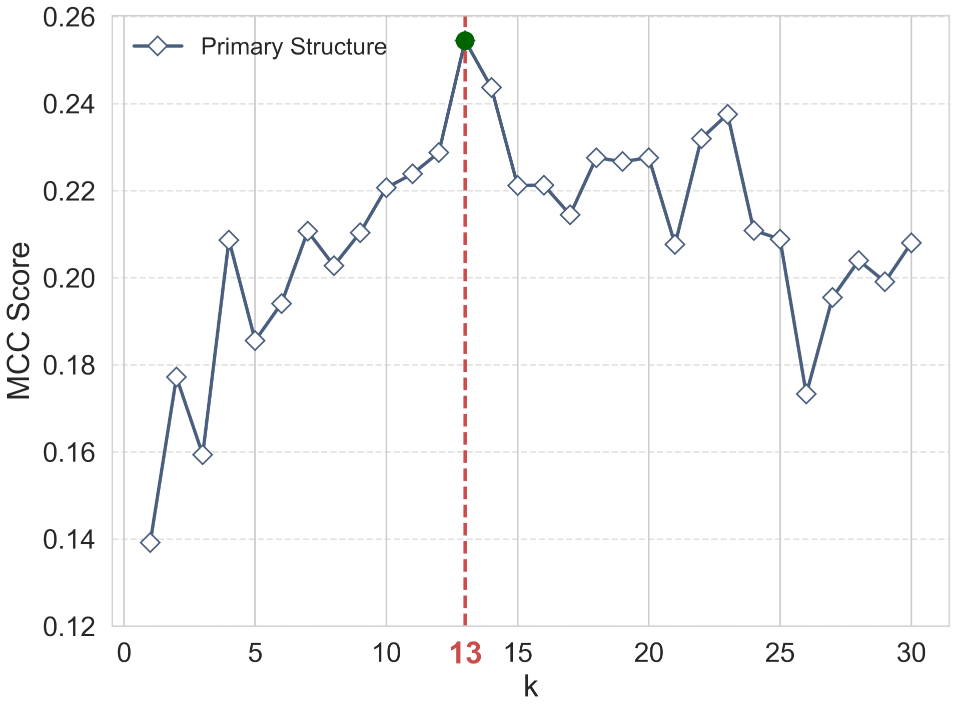

Fig. S1: The MCC values of MVRBind evaluated under different k values in the primary structural graph.

We replaced the GCN with a variety of alternative graph neural networks, including GraphSAGE, GATv2, k-GNNs, and TAGCN. Table S2 shows performance across test splits. GCN consistently outperformed others, likely due to better generalization and reduced overfitting on limited data.

Table S2: Comparison of different graph convolutional models.

Model	Test18	Apo test	Conformational test
GraphSAGE	0.728	0.746	0.629
GATv2	0.735	0.696	0.572
k-GNNs	0.732	0.749	0.581
GATv2Conv	0.722	0.712	0.616
TAGCN	0.730	0.738	0.551
GCN (Ours)	0.745	0.756	0.660

3 Details of self-attention application in multi-scale prediction module

The four scale-specific representations $Z_{\text{Node}}, Z_{\text{NA}}, Z_{\text{Loc}}, Z_{\text{Glob}}$ for each nucleotide are processed through a self-attention mechanism to capture the relevant dependencies and relationships at each scale. Given the four scale-specific representations, we first create a combined input matrix $Z_{\text{input}} \in R^{4\times d}$, where each row corresponds to one of the four representations ($Z_{\text{Node}}, Z_{\text{NA}}, Z_{\text{Loc}}, Z_{\text{Glob}}$) with dimensionality d. To apply self-attention, these representations are transformed into queries Q, keys K, and values V by applying linear projections to the input matrix E_{input}:

$$Q = Z_{\text{input}} W_Q, \quad K = Z_{\text{input}} W_K, \quad V = Z_{\text{input}} W_V. \tag{1}$$

Here, $W_Q, W_K, W_V \in R^{d\times d}$ are learnable weight matrices for the queries, keys, and values, respectively. These matrices enable the model to learn the most relevant representations for computing the attention scores. Next, we calculate the attention scores by computing the scaled dot-product between the queries Q and keys K. Specifically, the attention score A is given by:

$$A = \text{softmax}\left(\frac{QK^T}{\sqrt{d}}\right). \tag{2}$$

Here, $A \in R^{4\times 4}$ is the attention matrix, which represents the relative importance between different scales. The softmax function ensures that the attention scores sum to 1 across each row, producing a probability distribution for each scale's interaction with others. Once the attention scores are computed, they are applied to the values V to produce the self-attention-enhanced representations for each scale. The weighted summation of the values using the

attention scores is computed as follows:

$$Z_{\text{a}} = AV. \tag{3}$$

Here, $Z_{\text{a}} \in R^{4\times d}$ represents the self-attention-enhanced representations for each scale. These enhanced representations capture the relationships and dependencies between the different scales, allowing the model to focus on the most informative features. The self-attention-enhanced representations for each scale are denoted as: $Z^{\text{a}}_{\text{Node}}, Z^{\text{a}}_{\text{NA}}, Z^{\text{a}}_{\text{Loc}}, Z^{\text{a}}_{\text{Glob}}$. Each of these vectors contains the enhanced features for the corresponding scale, taking into account the interactions with the other scales. These representations are then used as input for the next step, where they are combined via average pooling to form the final multi-scale feature representation.

4 Binding sites prediction without experimental structures

Due to the limited availability of experimentally resolved RNA tertiary structures, it is necessary to predict binding sites for RNAs with unknown structures. To ensure a fair comparison with previous studies, we utilized RNAComposer [6] to predict the tertiary structures of the Test18 dataset and subsequently performed binding site predictions on these predicted structures. The results, summarized in Table S3, demonstrate that MVRBind outperforms existing methods across all evaluation metrics.

Table S3: Model comparison for predicted RNA structures on Test18 using RNAComposer.

**Model**	**Precision**	**Recall**	**MCC**	**AUC**
Rsite	0.403	0.130	0.047	0.515
RNAsite	<u>0.513</u>	0.372	<u>0.209</u>	<u>0.685</u>
RBind	0.373	0.106	0.023	0.507
RLBind	0.442	0.294	0.116	0.630
Rnet	0.448	0.231	0.106	0.619
Ours (w/o sec. str.)	0.487	<u>0.386</u>	0.190	0.669
**Ours**	**0.518**	**0.401**	**0.225**	**0.700**

To further analyze the relationship between the accuracy of tertiary structure predictions and binding site prediction performance, we computed the Pearson correlation coefficient between the RMSD of the predicted structures and the precision of the predicted binding sites, obtaining a correlation of 0.7173. While this correlation suggests that structural quality influences prediction performance, MVRBind remains effective by leveraging multi-view

graph construction, which ensures robust binding site identification even in the presence of structural deviations.

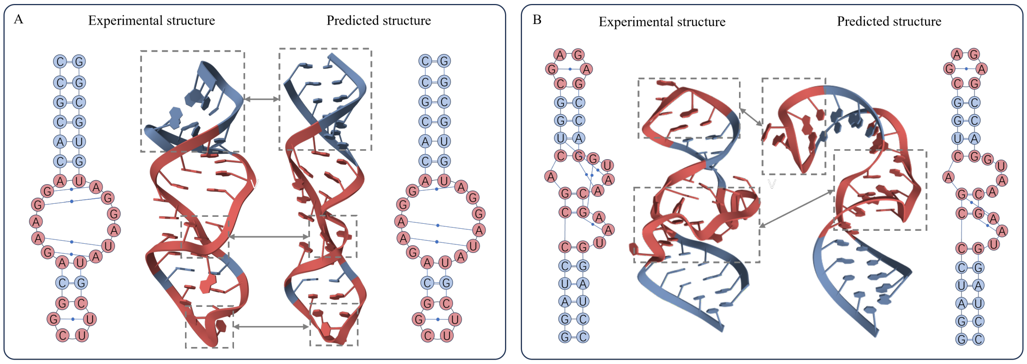

Fig. S2: The tertiary and secondary structures of the predicted models and the tertiary and secondary structures determined experimentally. A shows the RNA with PDB ID 1FMN, predicted using RNAComposer, and B shows the RNA with PDB ID 1AJU, predicted using AlphaFold3.

The robustness of our model in predicting binding sites for RNAs with unknown tertiary structures can be attributed to the multi-view graph construction, which facilitates effective information propagation. As illustrated in Fig. S2A, for the RNA with PDB ID: 1FMN, the predicted tertiary structure shows an RMSD of 7.14 Å, with notable deviations at both the 5' and 3' ends. However, the fundamental secondary structure motifs remain intact, particularly in the central binding site region, which preserves its structural integrity. This preservation of critical motifs enhances the model's ability to robustly predict binding sites, even in the absence of experimentally determined structures. To further verify this, we removed the secondary structure graph from the model and re-predicted the binding sites. As shown in the Table S3, the model's performance significantly declined, which further demonstrates that multi-view graph construction allows for accurate structural prediction within a certain margin of error.

Additionally, as depicted in Fig. S3, we observed instances where the predictions of the tertiary structure exhibited substantial inaccuracy, with RMSD values surpassing 20 Å. Further analysis revealed that these RNAs exist in PDB not as isolated single strands but rather as components of multi-chain complexes, where their structures are influenced by interactions with other RNA or protein chains. Since RNAcomposer only predicts the tertiary structure of a single RNA sequence, these interactions were not accounted for, leading to poor structural predictions.

To address this limitation, we employed AlphaFold3 [7] to predict the tertiary structures of the Test18 RNAs, incorporating a more comprehensive modeling of RNA structures. The predicted RMSD values, as shown in the Fig. S4, are lower than those obtained using RNA-

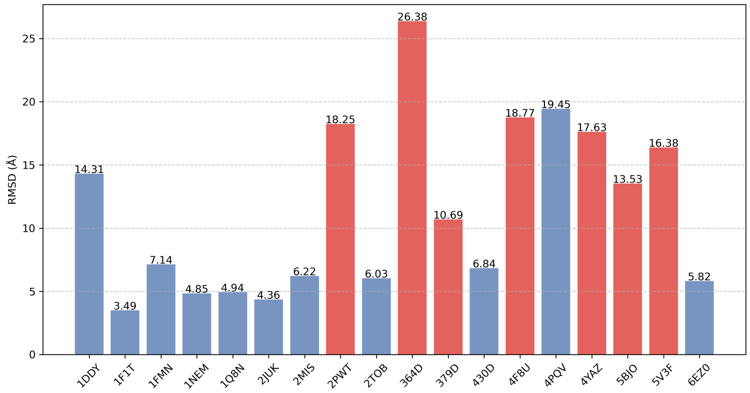

Fig. S3: The RMSD between the RNA structure predicted by RNAComposer and the experimentally determined RNA structure. Red represents interchain interactions in multi-chain RNAs, while blue indicates the absence of such interactions.

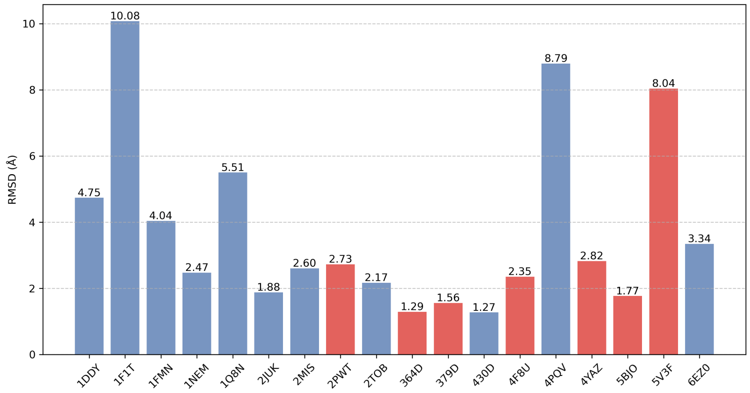

Fig. S4: The RMSD between the RNA structure predicted by AlphaFold3 and the experimentally determined RNA structure. Red represents interchain interactions in multi-chain RNAs, while blue indicates the absence of such interactions.

Composer, particularly in the case of multi-chain complexes. The prediction results are shown in Fig. S2B. Notably, even for the most poorly predicted RNA structure (PDB ID: 1AJU, RMSD = 10.08Å), the secondary structure motifs remained largely preserved, despite some deviations in non-canonical base pairings. Since small-molecule binding sites are often located within these motifs, the model remained capable of accurately predicting binding sites. We further performed binding site predictions on the AlphaFold3-generated structures, and the results, shown in the Fig. S5, indicate a slight decrease in precision. The AUC was 0.73, representing only a 2% drop compared to predictions using experimentally resolved structures.

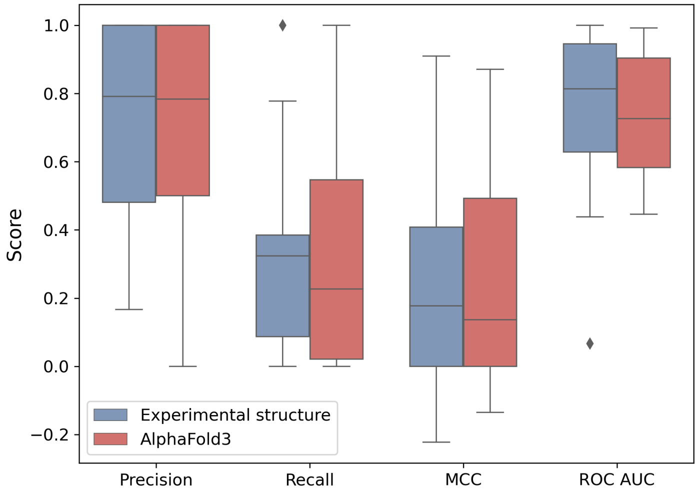

Fig. S5: Binding site prediction on AlphaFold3 predicted and experimentally resolved structures.

These findings further demonstrate that MVRBind maintains robust performance in binding site prediction, even for structurally flexible RNAs. The preservation of essential secondary structure motifs plays a crucial role in maintaining prediction accuracy, highlighting the model's ability to generalize effectively to RNAs with unknown tertiary structures.

5 Ablation study on Apo Test and Conformational Test

To systematically evaluate the contribution of different components in MVRBind to RNA binding site prediction, we conducted ablation studies on both the Apo test set and the Conformational test set. The model variants are categorized into three groups to assess the impact of structural information, feature representations, and multi-scale fusion mechanisms.

The first category examines the role of structural information by removing the graph representation at each scale. Specifically, we tested MVRBind without the primary structure graph (w/o prim. str.), MVRBind without the secondary structure graph (w/o sec. str.), and MVRBind without the tertiary structure graph (w/o ter. str.). These variants assess the extent to which structural connectivity at different levels contributes to accurate binding site predictions. The second category investigates the effect of feature representations by eliminating structure-derived features. The tested variants include MVRBind without primary structure features (w/o prim. feat.), MVRBind without secondary structure features (w/o sec. feat.), and MVRBind without tertiary structure features (w/o ter. feat.). This analysis pro-

Table S4: Results of ablation study on Apo and Conformational tests.

Models	Apo Test				Conformational Test			
	Precision	Recall	MCC	AUC	Precision	Recall	MCC	AUC
MVRBind (w/o prim. str.)	0.275	0.105	0.093	0.665	0.515	0.213	0.054	0.594
MVRBind (w/o sec. str.)	0.296	0.105	0.103	0.634	0.500	0.011	0.008	0.611
MVRBind (w/o ter. str.)	0.258	0.105	0.084	0.604	0.554	0.161	0.075	0.580
MVRBind (w/o prim. feat.)	0.157	0.039	0.010	0.622	0.577	0.287	0.128	0.615
MVRBind (w/o sec. feat.)	0.250	0.118	0.160	0.614	0.503	0.242	0.047	0.632
MVRBind (w/o ter. feat.)	0.277	0.131	0.106	0.688	0.527	0.236	0.069	0.614
MVRBind (w/o msf)	0.285	0.131	0.110	0.605	0.576	0.283	0.126	0.644
Ours	0.466	0.184	0.228	0.756	0.632	0.296	0.181	0.660

vides insights into the significance of structural embeddings at different scales. The third category evaluates the necessity of the multi-scale fusion mechanism, using a variant termed MVRBind without multi-scale fusion (w/o msf). This experiment aims to determine whether the integration of multi-scale information enhances model performance.

The results of the ablation studies on the Apo and Conformational test sets are presented in Table S4, which demonstrates the impact of removing various structural and feature components on the RNA binding site prediction performance. Specifically, the removal of any individual component led to a decline in performance across both test scenarios, underscoring the critical role of multi-scale structural information and the integration of fusion mechanisms in enhancing the robustness of RNA binding site prediction. These findings highlight the necessity of maintaining a comprehensive model that accounts for diverse structural and feature representations to achieve optimal prediction accuracy.

6 Similarity analysis between 8R62 and the training set

To address concerns regarding potential overlap between the test RNA (8R62) and the training set, we conducted a comprehensive analysis of both structural and sequence similarity between 8R62 and all RNAs in the training dataset. This was essential to ensure the fairness of the evaluation and to demonstrate that the model's performance on 8R62 reflects genuine generalization. For structural comparison, we utilized TM-align to compute TM-scores between 8R62 and each RNA in the Train60 dataset. As shown in Fig. S6, the majority of RNAs had TM-scores around 0.14, indicating very low structural similarity. Only two RNAs had TM-scores of 0.363 and 0.388, which remain well below the commonly accepted threshold of 0.5 for significant global fold similarity [8]. These results suggest that 8R62 does not share a similar overall structure with any RNA in the training set.Sequence similarity was assessed

using MMseqs2 clustering with a sequence identity threshold of 30%.

Table S5: MMseqs2-based RNA clustering at 30% sequence identity. The red-colored cluster and members (Cluster 48: 8R62) highlight the RNA forming a singleton cluster, indicating no significant sequence similarity to the training set.

Cluster	Members	Cluster	Members	Cluster	Members
Cluster 1	3GX3	Cluster 2	3MEI	Cluster 3	3OXE
Cluster 4	1EHT	Cluster 5	3Q3Z	Cluster 6	3Q50
Cluster 7	1FYP, 1BYJ	Cluster 8	3SKT	Cluster 9	1HR2
Cluster 10	3TZR	Cluster 11	1LVJ, 1AJU, 1ARJ, 1QD3, 1UTS, 1UUD, 2L8H	Cluster 12	3VRS
Cluster 13	1NBK	Cluster 14	4FRG	Cluster 15	1NTB
Cluster 16	4K31	Cluster 17	4LVX	Cluster 18	1RAW
Cluster 19	4MEG	Cluster 20	1TOB	Cluster 21	4MGM
Cluster 22	4NYB	Cluster 23	4PCJ	Cluster 24	1Y90
Cluster 25	4QJH	Cluster 26	1YLS	Cluster 27	4QLM
Cluster 28	1YRJ	Cluster 29	4RGE	Cluster 30	4XW7
Cluster 31	2G5K	Cluster 32	5D5L	Cluster 33	2KGP, 1EI2
Cluster 34	5DH8	Cluster 35	2KTZ	Cluster 36	5FJ1
Cluster 37	5J02	Cluster 38	2LWK	Cluster 39	5O69
Cluster 40	2MXS	Cluster 41	5U3G	Cluster 42	2NOK
Cluster 43	5VCI	Cluster 44	6FZ0	Cluster 45	2QUW
Cluster 46	6HAG	Cluster 47	2YIE	Cluster 48	8R62
Cluster 49	3BNQ	Cluster 50	3C44, 2FCY	Cluster 51	3FU2

As shown in Table S5, under this criterion, 8R62 was assigned to a singleton cluster, indicating that it shares less than 30% sequence identity with any RNA used for training. This confirms the absence of notable sequence-level overlap.

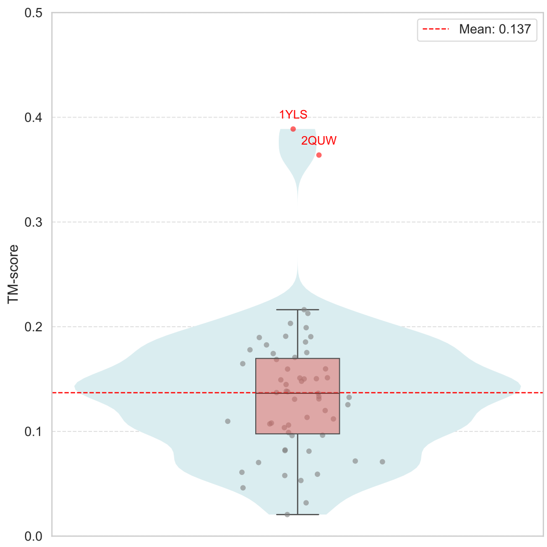

Fig. S6: Distribution of TM-scores between 8R62 and RNAs in the Train60 dataset. The two highest scores (0.363 and 0.388) are well below the 0.5 threshold for structural similarity.

To further eliminate the possibility that the model's performance might be influenced by the two structurally closer RNAs (with TM-scores of 0.363 and 0.388), we removed these RNAs from the training set and retrained the model. Evaluation on 8R62 using the retrained model yielded consistent results, reinforcing that the model does not rely on structurally or sequentially similar examples to make accurate predictions on this RNA.

Taken together, these results confirm that 8R62 is structurally and sequentially dissimilar to the RNAs in the training set, and that the model's performance reflects a true generalization capability rather than memorization or overfitting. This analysis provides strong support for the validity of the case study and the robustness of the model.

7 Error analysis on apo RNA structures

To better understand the limitations of our model, particularly in the absence of ligand structural cues, we conducted a localized error analysis on representative apo RNA samples. The goal was to investigate where false predictions are most likely to occur and assess their structural context. As shown in Fig. S7, the majority of false negatives (yellow nodes) and false positives (blue nodes) are concentrated in internal loop regions. These internal loops are typically solvent-exposed, flexible, and capable of forming diverse tertiary interactions. Compared to stems or other structured regions, internal loops tend to form relatively large cavities with irregular shapes and orientations. This poses challenges for precise residue-level prediction of ligand binding sites. Interestingly, although the model sometimes mislocalizes specific residues, it frequently identifies the correct broader cavity or binding pocket. In such cases, predicted binding residues may be slightly displaced due to the conformational plasticity of the loop or the lack of explicit ligand geometry during prediction. These deviations are particularly common in regions that can adopt alternative local structures or host transient interactions. Despite these local inaccuracies, the model remains robust in capturing the overall spatial location of binding regions.

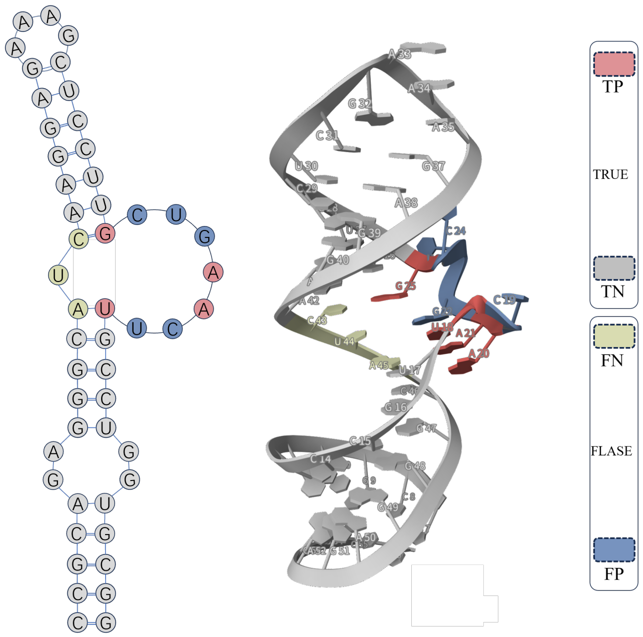

Fig. S7: Error analysis on representative apo RNA structures. False negatives (yellow) and false positives (blue) frequently occur in flexible internal loops. True positives are shown in red, and true negatives are shown in gray.

8 Robustness to predicted structure quality

To address concerns regarding the use of predicted RNA structures and potential error propagation in the MVRBind pipeline, we conducted analyses to evaluate how the model responds to structural inaccuracies in tertiary and secondary structure inputs. First, we examined how MVRBind performs when provided with tertiary structures that exhibit high root-mean-square deviation (RMSD) from experimental references. We found that MVRBind remains moderately robust in cases where the global fold and canonical base-pairing are preserved, even if local geometries deviate. However, when predicted structures exhibit severe distortions—such as collapsed helices or misoriented domains—performance, particularly precision, is noticeably reduced. Since AlphaFold3 provides per-residue confidence estimates (pLDDT), we recommend filtering out low-confidence models or regions prior to using MVRBind, which can mitigate the risk of false predictions from structurally unreliable inputs. Second, we explored whether MVRBind is suitable for use with predicted RNA models when experimentally resolved structures are not available. Empirically, MVRBind performs reliably on predicted structures generated by RNAComposer or AlphaFold3, provided that they maintain realistic folds and plausible base-pairing. Because MVRBind does

not perform structure refinement, we encourage users to apply basic quality control and confidence assessment before inputting predicted models into the pipeline. Third, to evaluate MVRBind's sensitivity to errors in secondary structure predictions, we conducted controlled perturbation experiments on RNA secondary structure graphs. Specifically, we randomly introduced noise into base-pairing edges and node features at various levels. As shown in Fig. S8, MVRBind retains stable average AUC performance on the test set when the noise ratio is below 10%. This robustness arises from its multi-modal design, which integrates sequence and 3D geometry to compensate for local secondary structure inaccuracies. Beyond this threshold, performance begins to degrade more substantially, highlighting the importance of reasonably accurate secondary structure inputs.

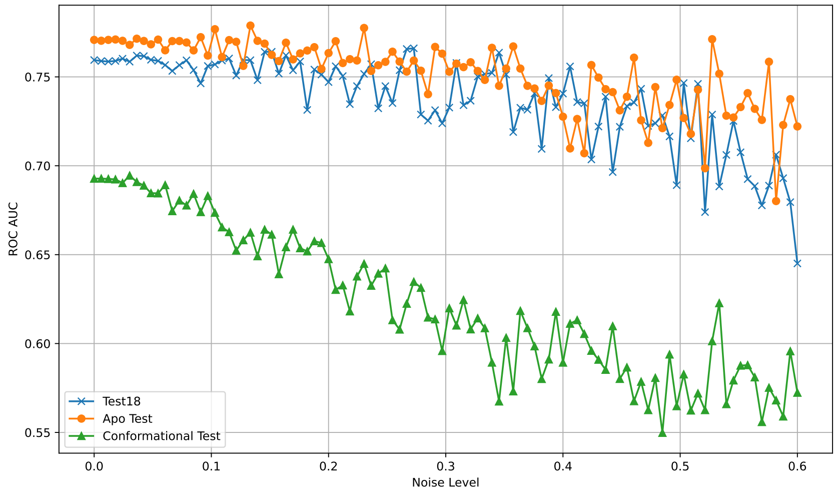

Fig. S8: Robustness of MVRBind to noise in secondary structure inputs. Line plot showing the average AUC of MVRBind on the test set under varying levels of noise added to the secondary structure graph.

9 Comprehensive Evaluation Metrics on Benchmark Datasets

Table S6: Performance comparison of different models on Set1–Set4 (HARIBOSS) using Precision, Recall, MCC, and ROC AUC as evaluation metrics.

Model	Set1				Set2				Set3				Set4			
	Precision	Recall	MCC	AUC	Precision	Recall	MCC	AUC	Precision	Recall	MCC	AUC	Precision	Recall	MCC	AUC
RLBind	0.302	0.260	0.125	0.712	0.278	0.140	0.147	0.711	0.178	0.060	0.050	0.661	0.259	0.280	0.204	0.601
Ret	0.364	0.295	0.188	0.731	0.178	0.034	0.044	0.717	0.204	0.185	0.112	0.673	0.212	0.047	0.066	0.657
RNABind	0.300	0.290	0.232	0.757	0.266	0.108	0.055	0.707	0.230	0.113	0.032	0.688	0.232	0.216	0.144	0.685
RNABERT+MLP	0.360	0.292	0.184	0.701	0.158	0.198	0.091	0.680	0.147	0.112	0.047	0.528	0.163	0.250	0.116	0.611
RNAErnie+MLP	0.376	0.164	0.142	0.621	0.317	0.198	0.198	0.687	0.129	0.027	0.015	0.573	0.227	0.059	0.080	0.597
RNA-FM+MLP	0.411	0.260	0.207	0.745	0.260	0.087	0.108	0.701	0.165	0.023	0.026	0.606	0.203	0.037	0.056	0.576
**Ours**	**0.411**	**0.308**	**0.306**	**0.785**	**0.330**	**0.202**	**0.207**	**0.740**	**0.266**	**0.270**	**0.188**	**0.697**	**0.272**	**0.341**	**0.220**	**0.693**

As shown in Table S6, the performance of various RNA–small molecule binding site prediction models is evaluated on four datasets (Set1 to Set4) from the HARIBOSS [9] benchmark, constructed using different data partitioning strategies. Metrics including Precision, Recall, MCC, and AUC are reported. Our model consistently achieves the best results across all datasets and metrics, particularly in MCC and AUC, indicating superior predictive accuracy and generalization.

Table S7: Performance comparison across different test sets. Rsite predicts binding sites based on sequence-derived secondary structure. Since all conformations in the Conformational test set share the same RNA sequence, Rsite yields identical predictions and is therefore not applicable to this evaluation.

Models	Test18				Apo Test				Conformational Test			
	Precision	Recall	MCC	AUC	Precision	Recall	MCC	AUC	Precision	Recall	MCC	AUC
Rsite	0.394	0.126	0.040	0.513	0.224	0.171	0.085	0.538	0.465	0.131	0.027	0.509
Rsite2	0.333	0.184	0.006	0.497	0.167	0.105	0.025	0.510	-	-	-	-
RNAsite	0.550	0.159	0.147	0.710	0.368	0.092	0.126	0.703	0.508	0.263	0.007	0.592
RBind	0.611	0.159	0.179	0.554	0.224	0.145	0.077	0.532	0.569	0.145	0.068	0.519
RLBind	0.632	0.320	0.277	0.719	0.200	0.013	0.016	0.577	0.597	0.109	0.076	0.398
Rnet	0.616	0.333	0.277	0.709	0.375	0.039	0.083	0.502	0.554	0.184	0.080	0.560
RNABind	0.463	0.341	0.263	0.717	0.266	**0.228**	0.144	0.657	0.442	0.274	0.018	0.532
RNABERT+MLP	0.569	0.338	0.243	0.682	0.215	0.144	0.070	0.696	0.472	0.285	0.142	0.599
RNAErnie+MLP	0.430	0.135	0.066	0.569	0.261	0.145	0.102	0.544	0.597	0.225	0.126	0.614
RNA-FM+MLP	0.435	0.275	0.105	0.591	0.203	0.144	0.061	0.586	0.475	0.183	0.015	0.593
MVRBind (*Ours*)	**0.645**	**0.342**	**0.351**	**0.745**	**0.466**	0.184	**0.228**	**0.756**	**0.632**	**0.296**	**0.181**	**0.660**

As shown in Table S7, model performance is compared across three test sets: Test18, Apo Test, and Conformational Test, with the same evaluation metrics. Notably, the sequence-based Rsite model produces identical predictions for conformations sharing the same RNA sequence, making it unsuitable for the Conformational Test. Together, these results highlight the robustness and improved accuracy of our approach across diverse RNA conformations and benchmark datasets.

References

[1] Sha Gong, Chengxin Zhang, and Yang Zhang. Rna-align: quick and accurate alignment of rna 3d structures based on size-independent tm-scorerna. *Bioinformatics*, 35(21): 4459–4461, 04 2019. ISSN 1367-4803.

[2] Hong Su, Zhenling Peng, and Jianyi Yang. Recognition of small molecule–rna binding sites using rna sequence and structure. *Bioinformatics*, 37(1):36–42, 2021.

[3] Kaili Wang, Renyi Zhou, Yifan Wu, and Min Li. Rlbind: a deep learning method to predict rna–ligand binding sites. *Briefings in Bioinformatics*, 24(1):bbac486, 2023.

[4] Haoquan Liu, Yiren Jian, Jinxuan Hou, Chen Zeng, and Yunjie Zhao. Rnet: a network strategy to predict rna binding preferences. *Briefings in Bioinformatics*, 25(1):bbad482, 2024.

[5] FP Panei, P Gkeka, and M Bonomi. Identifying small-molecules binding sites in rna conformational ensembles with shaman. *Nature Communications*, 15(1):5725, 2024.

[6] Marcin Biesiada, Katarzyna J Purzycka, Marta Szachniuk, Jacek Blazewicz, and Ryszard W Adamiak. Automated rna 3d structure prediction with rnacomposer. *RNA Structure Determination: Methods and Protocols*, pages 199–215, 2016.

[7] Josh Abramson, Jonas Adler, Jack Dunger, Richard Evans, Tim Green, Alexander Pritzel, Olaf Ronneberger, Lindsay Willmore, Andrew J Ballard, Joshua Bambrick, et al. Accurate structure prediction of biomolecular interactions with alphafold 3. *Nature*, 630 (8016):493–500, 2024.

[8] Jinrui Xu and Yang Zhang. How significant is a protein structure similarity with tm-score= 0.5? *Bioinformatics*, 26(7):889–895, 2010.

[9] Francesco P Panei, Rachel Torchet, Herve Menager, Paraskevi Gkeka, and Massimiliano Bonomi. Hariboss: a curated database of rna-small molecules structures to aid rational drug design. *Bioinformatics*, 38(17):4185–4193, 2022.
